# Generalized Legendre Transforms Have Roots in Information Geometry †

**DOI:** 10.3390/e28010044

**Published:** 2025-12-30

**Authors:** Frank Nielsen

**Affiliations:** Sony Computer Science Laboratories, 3-14-13 Higashi Gotanda, Shinagawa Ku, Tokyo 141-0022, Japan; frank.nielsen.x@gmail.com

**Keywords:** reverse-ordering and convex duality, legendre transform, information geometry, dually flat space and Hessian manifold, Bregman and Fenchel–Young divergences, affine and curvilinear coordinate systems

## Abstract

Artstein-Avidan and Milman [Annals of mathematics (2009), (169):661–674] characterized invertible reverse-ordering transforms in the space of lower, semi-continuous, extended, real-valued convex functions as affine deformations of the ordinary Legendre transform. In this work, we first prove that all those generalized Legendre transforms of functions correspond to the ordinary Legendre transform of dually corresponding affine-deformed functions. In short, generalized convex conjugates are ordinary convex conjugates of dually affine-deformed functions. Second, we explain how these generalized Legendre transforms can be derived from the dual Hessian structures of information geometry.

## 1. Introduction

Let R denote the field of real numbers and $\bar{R}=R\cup \left\{\pm \infty \right\}$ be the extended real line. A *m*-variate extended function $F:R^{m}\to \bar{R}$ is proper when its efficient domain Θ=dom(F):={θ:F(θ)<+∞} is non-empty and lower semi-continuous (lsc.) when its epigraph epi(F):={(θ,y):y≥F(θ),θ∈dom(F)} is closed with respect to the metric topology of $R^{m}$. We denote by $\Gamma_{0}$ (shorcut for $\Gamma_{0}\left(R^{m}\right)$) the space of of proper, lower, semi-continuous, extended, real-valued convex functions.

Consider the Legendre–Fenchel transform [1] (LFT) LF of a function *F*:

(1) $$\left(LF\right)\left(\eta \right):=\underset{\theta \in R^{m}}{\sup}\left\{\langle \theta ,\eta \rangle -F(\theta )\right\},$$

where $\langle v,v^{\prime }\rangle :=\sum_{i=1}^{m}v_{i}\;v_{i}^{\prime }$ denotes the Euclidean inner product. The function $F^{*}=LF$ is called the convex conjugate function. Some examples of convex conjugates are reported in Appendix B.

The Legendre transform originated in the work of Adrien-Marie Legendre [2] circa 1805. Legendre considered variational calculus problems and introduced the variable transform. The Legendre transform was later formalized by Hamilton [3] and Gibbs [4] as a way to switch between equivalent descriptions of a system by exchanging variables like position ↔ momentum or energy ↔ entropy. This variable duality principle induced by the Legendre transform turned out to be fundamental in many scientific fields, like thermodynamics [5] for relating thermodynamic potentials, quantum field theory [6] for connecting Lagrangian and Hamiltonian formulations (and underlying effective actions), economics for relating expenditure with utility functions [7], and convex optimization [8], where it is the cornerstone of duality theory in convex analysis.

The Moreau–Fenchel–Rockafellar theorem [9] states that when a function $F\in \Gamma_{0}$, its biconjugate function ${\left(F^{*}\right)}^{*}$ coincides with the function *F*: ${F^{*}}^{*}=F$. That is, the Legendre transform is an involutive transform of $\Gamma_{0}$. For general lsc. functions *F* (possibly non-convex), it is known that the biconjugate function ${\left(F^{*}\right)}^{*}:=L\left(LF\right)$ lower bounds the function *F* by the largest possible lsc. convex function ${\left(F^{*}\right)}^{*}\leq F$. This lower bound convexification property has been proven useful in machine learning [10].

This study was motivated by the fundamental result of Artstein-Avidan and Milman [11,12], who proved the following theorem:

**Theorem 1** 
([12], Theorem 7)**.** *Let T be an invertible transform such that the following are true:*

- *$F_{1}\leq F_{2}\Rightarrow TF_{2}\leq TF_{1}$;*
- *$TF_{1}\leq TF_{2}\Rightarrow F_{2}\leq F_{1}$.*

*Then, T is a generalized Legendre–Fenchel transform (GLFT), written canonically as*

(2) (TF)(η)=λ(LF)(Eη+f)+〈η,g〉+h,

*where λ>0, $E\in \mathrm{GL}(R^{m})$ (general linear group), $f,g\in R^{m}$, and h∈R.*

We note in passing that Fenchel [13,14] interpreted the graph of the Legendre transform of an *m*-variate function $F\in \Gamma_{0}$ as the polarity with respect to the paraboloid surface of $R^{m+1}$

$$P=\left\{\left(\theta ,y=\frac{1}{2}\sum_{i=1}^{m}\theta_{i}^{2}\right)\;:\;\theta \in R^{m}\right\}\in R^{m+1},$$

of the graph of *F*. This geometric polarity connection of the Legendre transform is all the more interesting since Böröczky and Schneider [15] characterized the duality of convex bodies in Euclidean spaces containing the origin in the interior into the same space [11].

In this work, we shall first prove that a generalized convex conjugate TF obtained by Equation (2) can always be expressed as the ordinary convex conjugate of a corresponding affine-deformed function. Namely, we shall show that

$$\left(TF\right)\left(\eta \right)=L\left(\lambda F(A\theta +b)+\langle \theta ,c\rangle +d)\right),$$

where $A\in \mathrm{GL}(R^{m})$, $b,c\in R^{m}$ and d∈R are defined according to E,f,g,h (details reported in Theorem 2). This equivalence result allows us to interpret the origin of the generalized Legendre transforms from the viewpoint of information geometry [16,17] and untangle the various degrees of freedom used when defining generalized Legendre transforms in Section 3.

## 2. Generalized Legendre Transforms as Ordinary Legendre Transforms

Consider a parameter

$$P=\left(\lambda ,A,b,c,d\right)\in P:=R_{>0}\times \mathrm{GL}\left(R^{m}\right)\times R^{m}\times R^{m}\times R,$$

and deform a function F(θ) by carrying out affine transformations of both the parameter argument and its output as follows:

(3) $$F_{P}\left(\theta \right):=\lambda \;F\left(A\theta +b\right)+\langle \theta ,c\rangle +d.$$

Those affine deformations preserve convexity:

**Property 1** 
(Convexity-preserving affine deformations)**.** *Let $F\in \Gamma_{0}$**.** Then, $F_{P}$ belongs to $\Gamma_{0}$ for all P∈P.*

**Proof.**  Let us check the convexity of $F_{P}$:

$$\alpha F_{P}\left(\theta_{1}\right)+\left(1-\alpha \right)F_{P}\left(\theta_{2}\right)=\lambda \left(\alpha F\left({\bar{\theta }}_{1}\right)+\left(1-\alpha \right)F\left({\bar{\theta }}_{2}\right)\right)+\langle \alpha \theta_{1}+\left(1-\alpha \right)\theta_{2}),c\rangle +d,$$

where ${\bar{\theta }}_{1}:=A\theta_{1}+b$ and ${\bar{\theta }}_{2}:=A\theta_{2}+b$. Since *F* is convex, we have $\alpha F\left({\bar{\theta }}_{1}\right)+\left(1-\alpha \right)F\left({\bar{\theta }}_{2}\right)\leq F\left(\alpha {\bar{\theta }}_{1}+\left(1-\alpha \right){\bar{\theta }}_{2}\right)$. Let ${\bar{\theta }}_{\alpha }^{\prime }:=\alpha {\bar{\theta }}_{1}+\left(1-\alpha \right){\bar{\theta }}_{2}=A{\bar{\theta }}_{\alpha }^{\prime }+b$ with $\theta_{\alpha }^{\prime }:=\alpha \theta_{1}+\left(1-\alpha \right)\theta_{2}$. We obtain

$$\begin{array}{ccc} \alpha F_{P}\left(\theta_{1}\right)+\left(1-\alpha \right)F_{P}\left(\theta_{2}\right) & \leq & \underset{=F_{P}\left(\alpha \theta_{1}+\left(1-\alpha \right)\theta_{2}\right)}{\underset{︸}{\lambda F\left(A{\bar{\theta }}_{\alpha }^{\prime }+b\right)+\langle \theta_{\alpha }^{\prime },c\rangle +d}}, \end{array}$$

hence proving that $F_{P}$ is convex. Since the lower semi-continuous property is ensured (i.e., the epigraph of $F_{P}$ is closed), we conclude that $F_{P}\in \Gamma_{0}$. □

Next, let us express the convex conjugate of a function $F_{P}$ according to the ordinary convex conjugate $F^{*}=LF$:

**Proposition 1** 
(LFT of an affine-deformed function)**.** *The Legendre transform of $F_{P}$ with P=(λ,A,b,c,d)∈P is*

$${\left(F_{P}\right)}^{*}={\left(F^{*}\right)}_{P^{⋄}}$$

*where*

(4) $$P^{⋄}:=\left(\lambda ,\frac{1}{\lambda }A^{-1},-\frac{1}{\lambda }A^{-1}c,-A^{-1}b,\langle b,A^{-1}c\rangle -d\right)\in P.$$

*That is, we have $L\left(F_{P}\right)={\left(LF\right)}_{P^{⋄}}$.*

**Proof.**  Let $\Gamma_{1}\subset \Gamma_{0}$ be defined as the class of differentiable, strictly convex Legendre-type functions [18] (see Appendix A). In general, given a function $F\left(\theta \right)\in \Gamma_{1}$, we proceed as follows to calculate its convex conjugate. First, we find the inverse function ${\left(\nabla F\right)}^{-1}$ of its gradient ∇F to obtain the gradient of the convex conjugate $\nabla F^{*}={\left(\nabla F\right)}^{-1}$. Then, we have

$$F^{*}\left(\eta \right)=\langle \theta ,\eta \rangle -F\left(\theta \right)=\langle {\left(\nabla F\right)}^{-1}\left(\eta \right),\eta \rangle -F\left({\left(\nabla F\right)}^{-1}\left(\eta \right)\right).$$

For parameter P=(λ,A,b,c,d), consider the strictly convex and differentiable function $F_{P}\left(\theta \right)=\lambda F\left(A\theta +b\right)+\langle c,\theta \rangle +d$ for an invertible matrix A∈GL(d,R), vectors $b,c\in R^{d}$, and scalars d∈R and $\lambda \in R_{>0}$. The gradient of $F_{P}$ is

$$\eta =\nabla F_{P}\left(\theta \right)=\lambda A^{⊤}\nabla F\left(A\theta +b\right)+c.$$

We denote by $G=F^{*}$ and $G_{P}={\left(F_{P}\right)}^{*}$ the Legendre convex conjugates of *F* and $F_{P}$, respectively. By solving the equation $\nabla F_{P}\left(\theta \right)=\eta$, we find the reciprocal gradient $\theta \left(\eta \right)=\nabla G_{P}\left(\eta \right)$:

$$\nabla G_{P}\left(\eta \right)=A^{-1}\;\nabla G\left(\frac{1}{\lambda }A^{-⊤}\left(\eta -c\right)\right)-b.$$

Therefore, the Legendre convex conjugate is obtained as follows:

$$\begin{array}{ccc} G_{P}\left(\eta \right) & = & \langle \eta ,\nabla G_{P}\left(\eta \right)\rangle -F_{P}\left(\nabla G_{P}\left(\eta \right)\right),\\ & = & \lambda^{\prime }\;G\left(A^{\prime }\eta +b^{\prime }\right)+\langle c^{\prime },\eta \rangle +d^{\prime }, \end{array}$$

where

$$\begin{array}{ccc} \lambda^{\prime } & = & \lambda ,\\ A^{\prime } & = & \frac{1}{\lambda }A^{-1},\\ b^{\prime } & = & -\frac{1}{\lambda }A^{-1}c,\\ c^{\prime } & = & -A^{-1}b,\\ d^{\prime } & = & \langle b,A^{-1}c\rangle -d. \end{array}$$

Hence, it follows that we have $P^{⋄}=\left(\lambda ,\frac{1}{\lambda }A^{-1},-\frac{1}{\lambda }A^{-1}c,-A^{-1}b,\langle b,A^{-1}c\rangle -d\right)\in P.$ □

Let us check that the (diamond) ⋄ operator on affine deformation parameters is an involution:

**Proposition 2** 
(⋄ involution)**.** *The parameter transformation $P^{⋄}$ is an involution, where ${\left(P^{⋄}\right)}^{⋄}=P$.*

**Proof.**  Let P=(λ,A,b,c,d) and $P^{⋄}=\left(\lambda^{\prime },A^{\prime },b^{\prime },c^{\prime },d^{\prime }\right)$ with

$$\begin{array}{ccc} \lambda^{\prime } & = & \lambda ,\\ A^{\prime } & = & \frac{1}{\lambda }A^{-1},\\ b^{\prime } & = & -\frac{1}{\lambda }A^{-1}c,\\ c^{\prime } & = & -A^{-1}b,\\ d^{\prime } & = & \langle b,A^{-1}c\rangle -d. \end{array}$$

We check that ${P^{⋄}}^{⋄}=\left(\lambda^{\prime \prime },A^{\prime \prime },b^{\prime \prime },c^{\prime \prime },d^{\prime \prime }\right)=\left(\lambda ,A,b,c,d\right)=P$ component-wise as follows:

$$\begin{array}{cccccccc} & \lambda^{\prime \prime } & = & \lambda^{\prime } & = & \lambda ,\\ & A^{\prime \prime } & = & \frac{1}{\lambda }{A^{\prime }}^{-1} & = & \frac{1}{\lambda }{\left(\frac{1}{\lambda }A^{-1}\right)}^{-1} & = & A,\\ & b^{\prime \prime } & = & -\frac{1}{\lambda }{A^{\prime }}^{-1}c^{\prime } & = & -\frac{1}{\lambda }{\left(\frac{1}{\lambda }A^{-1}\right)}^{-1}\left(-A^{-1}b\right) & = & b,\\ & c^{\prime \prime } & = & -{A^{\prime }}^{-1}b^{\prime } & = & -{\left(\frac{1}{\lambda }A^{-1}\right)}^{-1}\left(-\frac{1}{\lambda }A^{-1}c\right) & = & c,\\ & d^{\prime \prime } & = & \langle b^{\prime },{A^{\prime }}^{-1}c^{\prime }\rangle -d^{\prime } & = & \langle -\frac{1}{\lambda }A^{-1}c,\frac{1}{\lambda }{\left(\frac{1}{\lambda }A^{-1}\right)}^{-1}\left(-A^{-1}b\right)\rangle -\langle b,A^{-1}c\rangle +d & = & d. \end{array}$$

□

The ⋄ involution confirms that the Legendre–Fenchel transform is an involution:

$${\left({\left(F_{P}\right)}^{*}\right)}^{*}=L\left(LF_{P}\right)=LF_{P^{⋄}}=F_{{\left(P^{⋄}\right)}^{⋄}}=F_{P}.$$

Let us now define the following notation to express the generalized Legendre convex conjugates:

**Definition 1** 
(Generalized Legendre–Fenchel convex conjugates [12])**.** *Let $L_{\lambda ,E,f,g,h}$ denote a generalized Legendre–Fenchel transform*

$$L_{\lambda ,E,f,g,h}F:=L_{P}F:=\lambda \left(LF\right)\left(E\eta +f\right)+\langle \eta ,g\rangle +h$$

*for the parameter P=(λ,E,f,g,h)∈P.*

Our result is that we can interpret those generalized Legendre–Fenchel transforms of Equation (2) as the ordinary Legendre transform on corresponding affine-deformed convex functions:

**Theorem 2.** 
For any $F\in \Gamma_{0}$, we have $L_{P}\left(F\right):={\left(F^{*}\right)}_{P}=L\left(F_{P^{⋄}}\right)$.

**Proof.**  By definition, $L_{P}F:={\left(LF\right)}_{P}$. Since $P={\left(P^{⋄}\right)}^{⋄}$ (Proposition 2), we have ${\left(LF\right)}_{P}={\left(LF\right)}_{{\left(P^{⋄}\right)}^{⋄}}$, and by using Proposition 1, we obtain ${\left(LF\right)}_{{\left(P^{⋄}\right)}^{⋄}}=L\left(F_{P^{⋄}}\right)$. To summarize, we have

$$L_{P}\left(F\right)={\left(LF\right)}_{P}={\left(LF\right)}_{{\left(P^{⋄}\right)}^{⋄}}=L\left(F_{P^{⋄}}\right).$$

□

That is, in plain words, the Artstein-Avidan–Milman generalized Legendre transforms [11,12] are ordinary Legendre transforms on affine-deformed functions evaluated on affine deformed arguments.

Next, we describe an information-geometric interpretation of Theorem 2 which casts light on the origin and meanings of the degrees of freedom used to define generalized Legendre transforms. Thus, understanding the ordinary Legendre transform from the information-geometric viewpoint allows one to understand how to recover the generalized Legendre transforms formalized axiomatically by Artstein-Avidan–Milman [11,12].

## 3. An Information-Geometric Interpretation of Generalized Legendre Transforms

We can interpret the fact that generalized Legendre convex conjugates are affine-deformed convex conjugates from the lens of information geometry [16]. Consider a smooth, strictly convex function $F:R^{m}\to \bar{R}$ of the space $\Gamma_{2}$ of twice-differentiable lsc. Legendre-type convex functions (with $\Gamma_{2}\subset \Gamma_{1}\subset \Gamma_{0}$). This function F(θ) defines a dually flat *m*-dimensional space [16] $\left(M,g,\nabla ,\nabla^{*}\right)$ which is a global chart manifold M={p} of points *p* equipped with dual Hessian structures [19] (g,∇) and $\left(g,\nabla^{*}\right)$. The primal Hessian structure is induced by a potential function ψ on *M* with a torsion-free, flat, affine connection ∇ such that ψ(p)=F(θ(p)), where (M,θ(·)) is the primal ∇-affine coordinate system. The dual Hessian structure is induced by a dual potential function ϕ on *M* with a dual torsion-free, flat, affine connection $\nabla^{*}$ such that $\phi \left(p\right)=F^{*}\left(\eta \left(p\right)\right)$, where (M,η(·)) is the dual $\nabla^{*}$-affine coordinate system. The Riemannian metric *g* can be expressed in the dual coordinate charts as $g\left(\theta \right)=\nabla^{2}F\left(\theta \right)$ or $g\left(\eta \right)=\nabla^{2}F^{*}\left(\eta \right)$, where $F^{*}$ is the convex conjugate. These two potential functions ψ and ϕ living on the manifold *M* satisfy the Fenchel–Young inequality:

(5) $$\psi \left(\theta \left(p\right)\right)+\phi \left(\eta \left(q\right)\right)\geq \sum_{i=1}^{m}\theta_{i}\left(p\right)\;\eta_{i}\left(q\right),$$

with equality if and only if p=q on *M*. The metric tensor *g* can be expressed as g=∇dψ or equivalently as $g=\nabla^{*}d\phi$, where *d* denotes the exterior derivative and ψ and ϕ are 0 forms.

Now, the ∇-affine coordinate system θ is defined up to an affine transformation. That is, if θ(·) is a ∇ coordinate system, then the coordinate system is $\bar{\theta }\left(\cdot \right)=A\theta \left(\cdot \right)+b$. However, once parameters *A* and *b* are fixed, it fully determines the dual $\nabla^{*}$ coordinate system η(·).

The potential function ϕ is also reconstructed by solving differential modulo equations an affine term 〈c,θ〉+d (e.g., see the proofs relying on Poincaré lemma in [16,19,20,21]). Fixing the degrees of freedom in the reconstruction of ψ will also fix the corresponding affine term in $F^{*}$ which expresses ψ. The dual potential functions ψ and ϕ on the manifold *M* are related by the fiberwise Legendre transform [22] in geometric mechanics.

Thus, the information geometry of dually flat spaces allows one to explain the decoupling of the interactions of the dual set of parameters (A,b,c,d) with (E,f,g,h). Lastly, the scalar parameter λ>0 is the degree of freedom obtained from the fact that if $\left(M,g,\nabla ,\nabla^{*}\right)$ is a dually flat space, then so is $\left(M,\lambda g,\nabla ,\nabla^{*}\right)$.

The Fenchel–Young inequality induced by the dual potential functions of Equation (5) defines a canonical divergence on a dually flat space that we term the dually flat Hessian divergence:

$$D_{g,\nabla ,}\left(p:q\right)=\psi \left(\theta \left(p\right)\right)+\phi \left(\eta \left(q\right)\right)-\sum_{i=1}^{m}\theta_{i}\left(p\right)\eta_{i}\left(q\right)\geq 0.$$

We have the following reference duality [23]: $D_{g,\nabla }\left(q:p\right)=D_{g,\nabla^{*}}\left(p:q\right)$.

The dually flat Hessian divergence can be expressed using the dual coordinate systems as a corresponding Fenchel–Young divergence defined by

$$Y_{F,F^{*}}\left(\theta :\eta^{\prime }\right):=F\left(\theta \right)+F^{*}\left(\eta^{\prime }\right)-\sum_{i=1}^{m}\theta_{i}\;\eta_{i}^{\prime }.$$

A Fenchel–Young divergence can also be expressed equivalently as dual Bregman divergences $B_{F}$ or $B_{F^{*}}$:

$$Y_{F,F^{*}}\left(\theta :\eta^{\prime }\right)=B_{F}\left(\theta :\theta^{\prime }\right)=B_{F^{*}}\left(\eta^{\prime }:\eta \right),$$

where $\theta^{\prime }=\nabla F^{*}\left(\eta^{\prime }\right)$ and η=∇F(θ) with

$$B_{F}\left(\theta :\theta^{\prime }\right)=F\left(\theta \right)-F\left(\theta^{\prime }\right)-\langle \theta -\theta^{\prime },\nabla F\left(\theta^{\prime }\right)\rangle .$$

Thus, the dually flat Hessian divergence can be expressed as dual Fenchel–Young divergences (using the fact that ${F^{*}}^{*}=F$) in the mixed θ/η coordinate systems as

(6) $$\begin{array}{ccc} D_{g,\nabla ,}\left(p:q\right) & = & Y_{F,F^{*}}\left(\theta \left(p\right):\eta \left(q\right)\right)=Y_{F^{*},F}\left(\eta \left(q\right):\theta \left(p\right)\right), \end{array}$$

(7) $$\begin{array}{ccc} & = & \frac{1}{\lambda }\;Y_{F_{P},F_{P^{⋄}}^{*}}\left(\bar{\theta }\left(p\right):\bar{\eta }\left(q\right)\right)=\frac{1}{\lambda }\;Y_{F_{P^{⋄}}^{*},F_{P}}\left(\bar{\eta }\left(q\right):\bar{\theta }\left(p\right)\right),\forall P\in P, \end{array}$$

This follows from the fact that $\psi \left(p\right)=F_{P}\left(\bar{\theta }\left(p\right)\right)=F\left(\theta \left(p\right)\right)$ and $\phi \left(q\right)=F_{P^{⋄}}^{*}\left(\bar{\eta }\left(q\right)\right)=F^{*}\left(\eta \left(q\right)\right)$ for any P∈P.

Thus, we can define an equivalence relation ∼ between the functions of $\Gamma_{2}$: $F∼\tilde{F}$ if and only if $\tilde{F}=F_{P}$ for some P∈P. Furthermore, a distance invariant under the Legendre transform [24] can be defined in the moduli space $\Gamma_{2}/∼$ of dually flat spaces.

Lastly, we may consider some curvilinear dual coordinate systems instead of the mutually orthogonal, dually affine coordinate systems. Let us transform the affine θ coordinate system into some arbitrary curvilinear coordinate system $\tilde{\theta }$. The dual affine η coordinate system transforms correspondingly into the curvilinear coordinate system $\tilde{\eta }$. The underlying geometric structures have been called the (u,v) structures by Amari [16,25] or the (ρ,τ) structures by Zhang [23]. The dual Bregman divergences or, equivalently, the dual Fenchel–Young divergences of the underlying dually flat space need to be addressed as undeforming arguments [26,27] $\tilde{\theta }$ or $\tilde{\eta }$. Since the inverse transform $\tilde{\theta }\to \theta$ can be interpreted as a representation function, the Bregman divergences addressed as undeforming $\tilde{\theta }$ arguments were called representational Bregman divergences in [26]. For example, the β divergences are Bregman divergences [28,29,30] which can be transformed by a nonlinear change of coordinates into α divergences [31]. Although the α divergences are Bregman divergences for any α on positive measures [26,27], α divergences are not Bregman divergences anymore when constrained on the probability simplex (except for the α=±1 case).

**Remark 1.** 
*In information geometry [16], and more generally in Hessian geometry [19], a Legendre-type $C^{3}$ convex function F(θ) of $\Gamma_{3}$ induces a dually flat space or manifold. The convex function F(θ) (potential function) may stem from several modeling sources. We may consider the following settings to build dually flat spaces:*

- *The cumulant function of a natural exponential family [16,32]: Let (X,Ω,μ) be a measurable space with a sample space X, σ algebra* Ω*, and a positive measure μ (e.g., counting or Lebesgue measure). An exponential family E is a set of probability measures $E=\left\{P_{\theta }\left(x\right)=\exp\left(\langle x,\theta \rangle -F\left(\theta \right)\right)\;d\mu \left(x\right)\;:\;\theta \in \Theta \right\}$, where*
  Θ={θ:F(θ)=log∫exp(〈x,θ〉)dμ(x)<∞},
  is the natural parameter space. Z(θ)=exp(F(θ)) is strictly log-convex [32], and hence F(θ)=logZ(θ) is strictly convex (and differentiable). In this case, the convex conjugate amounts to the negative Shannon or differential entropy [33] $F^{*}\left(\eta \right)=-S\left(p_{\theta }\right)=\int p_{\theta }\left(x\right)\log p_{\theta }\left(x\right)\;d\mu \left(x\right)$, where S(p)=−∫p(x)logp(x)dμ(x) is the Shannon entropy (when μ is the counting measure) or the differential entropy (when μ is the Lebesgue measure).
- *The negative Shannon entropy of a mixture family [16]: A mixture family M is a set of probability measures*
$$M=\left\{m_{\theta }\left(x\right)=\sum_{i=1}^{d-1}\theta_{i}p_{i}\left(x\right)+\left(1-\sum_{i=1}^{d-1}\theta_{i}\right)p_{0}\left(x\right)\right\},$$
  parameterized by a normalized positive weight vector $\theta \in \Theta =\Delta_{d}$ (the standard (d−1)-dimensional simplex) such that the functions $1,p_{0}\left(x\right),\ldots ,p_{d-1}\left(x\right)$ are linearly independent. The function $F\left(\theta \right)=-S\left(m_{\theta }\right)$ is strictly convex and differentiable (see [34] for a proof). In this case, the convex conjugate $F^{*}\left(\eta \right)=S^{\times }\left(p_{0},m_{\theta }\right)$ is the cross-entropy of $p_{0}\left(x\right)$ with the mixture $m_{\theta }\left(x\right)$, where $S^{\times }\left(p,q\right)=-\int p\left(x\right)\log q\left(x\right)\;d\mu \left(x\right)$.
- The characteristic function of a regular cone [19] (i.e., convex and pointed cone): Let K be a regular cone and $K^{*}=\cap_{\theta \in K}\left\{\eta \;:\;\langle \theta ,\eta \rangle \geq 0\right\}$ be its dual cone. We define $F\left(\theta \right)=\log\chi_{K}\left(\theta \right)$, where $\chi_{K}\left(\theta \right)$ is the characteristic function $\chi_{K}\left(\theta \right)=\int_{K^{*}}\exp\left(-\langle \theta ,\eta \rangle \right)\;d\eta$. One can further build generalized Wishart exponential families on the cones [35]. (Note that Massieu [36] introduced the concept of characteristic functions and their convex conjugates in thermodynamics in the 19th century.) See also the barrier functions (logarithms of characteristic functions) on convex cones for interior point methods [37].
- *The Guillemin potential of a (Delzant) polytope [38] which recovers the negative Shannon entropy when considering the standard simplex.*

## 4. Conclusions

To summarize, we first proved that the generalized Legendre transforms obtained from the reverse-ordering involutive transform axiomatization of [11,12] can be interpreted as the ordinary Legendre transform of corresponding affine-deformed functions. Second, we explained how these generalized Legendre transforms are merely different expressions of the well-known geometric Legendre transforms on dually flat spaces (Hessian manifolds with global charts).

**Figure 1:**
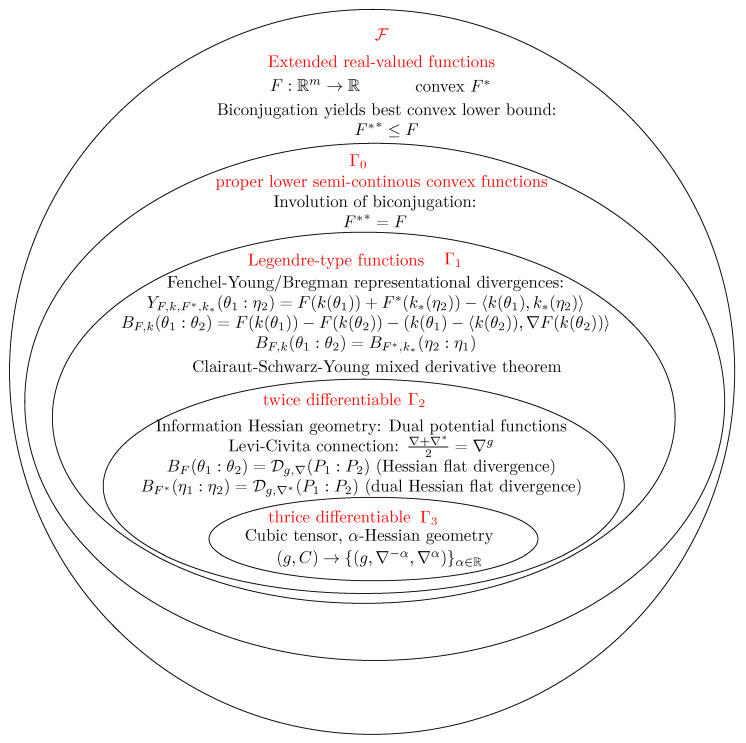
The ordinary Legendre transform for various classes of functions: relationships with Fenchel–Young and Bregman divergences, dually flat Hessian divergence, and α geometry in information geometry.

Figure 1 summarizes the various classes of convex functions used in this paper, with some of their key properties used to define information-geometric structures. In particular, the class of functions $\Gamma_{3}$ is thrice-differentiable Legendre-type lsc. convex functions (with $\Gamma_{3}\subset \Gamma_{2}\subset \Gamma_{1}\subset \Gamma_{0}$), which allows one to construct α geometry [16] from the Amari–Chentsov cubic tensor [17].

## Figures and Tables

**Figure A1:**
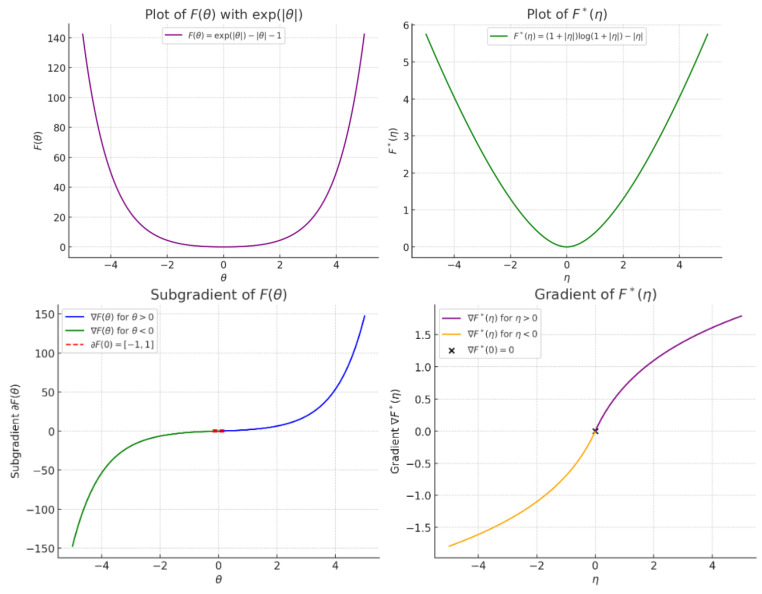
A pair $\left(F\left(\theta \right),F^{*}\left(\eta \right)\right)$ of conjugate functions (**top**) with their subgradients plotted (**bottom**). The function F(θ) is not differentiable at θ=0 and thus admits a subgradient ∂F(0) at θ=0. The function $F^{*}\left(\eta \right)$ is differentiable everywhere, and when θ≠0, we have $\nabla F^{*}={\left(\nabla F\right)}^{-1}$, visualized by rotating the xy axis 90 degrees).

## Data Availability

No new data were created or analyzed in this study. Data sharing is not applicable to this article.

## Appendix A. Legendre-Type Functions

Rockafellar [18] showed that there exist convex lsc. functions of $\Gamma_{0}$ with corresponding gradient domains that are not convex. For example, Rockafellar showed that the bivariate function $F\left(\theta \right)=F\left(\theta_{1},\theta_{2}\right)=\frac{1}{4}\left(\frac{\theta_{1}^{2}}{\theta_{2}}+\theta_{1}^{2}+\theta_{2}^{2}\right)$ defined on the upper plane domain $\Theta =R\times R_{>0}$ is strictly convex, but its gradient domain *H* is not convex. Thus, to rule out these functions, Rockafellar defined Legendre-type functions [18]:

**Definition A1** 
(Legendre-type function [18])**.** *A convex function $F:\Theta \subset R^{m}\to \bar{R}$ is of a Legendre type if the following are true:*

- Θ *is a non-empty open effective domain;*
- *F is strictly convex and differentiable on* Θ;
- *F becomes infinitely steep close to boundary points of its effective domain:*
  $$\forall \theta \in \Theta ,\forall \theta^{\prime }\in \partial \Theta ,\;\lim_{\lambda \to 0}\;\frac{d}{d\lambda }F\left(\lambda \theta +\left(1-\lambda \right)\theta^{\prime }\right)=-\infty .$$

When *F* is of a Legendre type, so is its convex conjugate $F^{*}$, and moreover the gradients of convex conjugates are reciprocal to each other such that $\nabla F^{*}={\left(\nabla F\right)}^{-1}$ and $\nabla F={\left(\nabla F^{*}\right)}^{-1}$. This property of reciprocal gradients obtained from the LFT of Legendre-type functions is strong since, in general, the implicit function theorem only guarantees local inversion of multivariate functions and not the existence of global inverse functions.

We shall denote by $\Gamma_{1}\left(\Theta \right)\subset \Gamma_{0}\left(\Theta \right)$ the subset of proper lsc. strictly convex and differentiable functions of a Legendre type defined on the open effective domain Θ. We define the Legendre–Fenchel transform of $L\left(\Theta ,F\right)=\left(H,F^{*}\right)$ where *H* is the gradient domain, and we call $F^{*}$ the convex conjugate of *F*. We have LL(Θ,F)=(Θ,F) for Legendre-type functions $F\in \Gamma_{1}\left(\Theta \right)$. The concept of a Legendre-type function is related to the concept of steep exponential families in statistics.

In general, convex conjugate pairs enjoy the following reverse-ordering property:

**Property A1** 
(Reverse-ordering)**.** *Let $F_{1}$ and $F_{2}$ in $\Gamma_{0}\left(\Theta \right)$. If $F_{2}\leq F_{1}$, then $LF_{2}\geq LF_{1}$. If $F_{2}\geq F_{1}$, then $LF_{2}\leq LF_{1}$.*

**Proof.** Assume that $F_{2}\leq F_{1}$, and let us prove that $F_{2}^{*}=LF_{2}\geq F_{1}^{*}=LF_{1}$:

$$\begin{array}{ccc} F_{1}^{*}\left(\eta \right) & = & \underset{\theta }{\sup}\left\{\langle \eta ,\theta \rangle -F_{1}\left(\theta \right)\right\},\\ & = & \langle \eta ,\nabla F^{-1}\left(\eta \right)\rangle -F_{1}\left(\nabla F^{-1}\left(\eta \right)\right),\\ & \leq & \langle \eta ,\nabla F^{-1}\left(\eta \right)\rangle -F_{2}\left(\nabla F^{-1}\left(\eta \right)\right),\\ & \leq & \underset{\theta }{\sup}\left\{\langle \eta ,\theta \rangle -F_{2}\left(\theta \right)\right\}=F_{2}^{*}\left(\eta \right). \end{array}$$

The case $F_{2}\geq F_{1}$ is similar to the case $F_{1}\geq F_{2}$ by exchanging the role of $F_{1}$ and $F_{2}$ (i.e., $F_{2}↔F_{1}$). □

## Appendix B. Some Examples of Convex Conjugates

**Example A1.** 

*Let F(θ)=〈a,θ〉+b be an affine function. Then, we have*

$$F^{*}\left(\eta \right)=\left\{\begin{array}{cc} -b & if\eta =a\\ +\infty & if\eta \neq a \end{array}\right.$$

*Let*

$$1_{A}\left(x\right)=\left\{\begin{array}{cc} 0 & ifx\in A\\ +\infty & ifx\notin A \end{array}\right.$$

*be the indicator function of a set A. Then, we can write $F^{*}=1_{\left\{a\right\}}-b$.*

**Example A2.** 

*Let F(θ)=|θ| for θ∈R. Then, we have the convex conjugate*

$$F^{*}\left(\eta \right)=1_{\left[-1,1\right]}\left(\eta \right)=\left\{\begin{array}{cc} 0 & |\eta |\leq 1,\\ +\infty & otherwise. \end{array}\right.$$

**Example A3.** 

*Consider F(θ)=expθ for Θ=R. We have*

$$F^{*}\left(\eta \right)=\left\{\begin{array}{cc} \eta \log\eta -\eta , & if\eta >0,\\ 0, & if\eta =0,\\ +\infty & if\eta <0. \end{array}\right.$$

*The convex conjugate is the scalar Shannon entropy function extended to positive real values.*

**Example A4.** 

*For p∈[1,+∞), let $F_{p}\left(\theta \right)=\frac{1}{p}{∥\theta ∥}^{p}$ for $\theta \in \Theta =R^{m}$. Then, we have $F_{p}^{*}\left(\eta \right):=F_{q}\left(\eta \right)$, where $\frac{1}{p}+\frac{1}{q}=1$. The powers p and q are called a conjugate pair of exponents. Notice that the Fenchel–Young inequality reduces to the Young’s inequality in the scalar case:*

$$F_{p}\left(\theta^{\prime }\right)+F_{q}\left(\eta \right)\geq \langle \theta^{\prime },\eta \rangle .$$

*Furthermore, by integrating Young’s inequality on both sides for η=g(x) and θ=f(x) for x∈X and $f\in L_{p}$ and $g\in L_{q}$ (Lebesgue spaces), we recover Hölder’s inequality:*

$$\frac{1}{p}{∥f∥}_{p}+\frac{1}{q}{∥g∥}_{q}\geq {∥fg∥}_{1}.$$

*In fact, we have the self-duality $F=F^{*}$ only for $F\left(\theta \right)=F_{2}\left(\theta \right)=\frac{1}{2}{∥\theta ∥}^{2}$.*

When a function $F\in \Gamma_{0}\left(\Theta \right)$ is not of the Legendre type, the Fenchel–Young inequality may be tight for several pairs $\left(\theta^{\prime },\eta \right)$ instead of a single pair $\left(\theta =\nabla F^{*}\left(\eta \right),\eta =\nabla F\left(\theta \right)\right)$. A subgradient η of F(θ) at $\theta_{0}$ satisfies

$$F\left(\theta \right)-F\left(\theta_{0}\right)\geq \langle \eta ,\theta -\theta_{0}\rangle .$$

The subdifferential $\partial_{\theta_{0}}F$ of *F* at $\theta_{0}$ is the set of subgradients. The subdifferential operator $\partial :R^{m}⇉R^{m}$ is multivalued:

$$\partial F\left(\theta \right)=\left\{\begin{array}{cc} \left\{\eta \;:\;F\left(\theta \right)-F\left(\theta_{0}\right)\geq \langle \eta ,\theta -\theta_{0}\rangle \right\}, & if\theta \in \Theta =\mathrm{dom}(F)\\ \emptyset & if\theta \notin \Theta . \end{array}\right.$$

The following example illustrates the LFT of a non-Legendre type function and the LFT of a Legendre-type function obtained by restricting the function to a specified domain.

**Example A5.** 

*Let F(θ):=exp(|θ|)−|θ|−1 be defined on Θ=R. Then, $F^{*}\left(\eta \right)=\left(1+|\eta |)\log(1+|\eta |)-|\eta \right|$, defined on H=R. These two functions F and $F^{*}$ are even and convex, and they form a conjugate pair (Figure A1). The function F(θ) is not differentiable at θ=0, and thus we have*

$$\partial F\left(\theta \right)=\left\{\begin{array}{cc} \nabla F(\theta )=\exp(\theta )-1, & \theta >0,\\ \partial F(0)=[-1,1], & \theta =0,\\ \nabla F(\theta )=-\exp(-\theta )+1, & \theta <0. \end{array}\right.$$

*The function $F^{*}\left(\eta \right)$ is differentiable everywhere, and we have*

$$\partial F^{*}\left(\eta \right)=\left\{\begin{array}{cc} \nabla F^{*}\left(\eta \right)=\log\left(1+\eta \right), & \eta \geq 0,\\ \nabla F^{*}\left(\eta \right)=-\log\left(1-\eta \right), & \eta \leq 0. \end{array}\right.$$

*For θ≠0, the gradients ∇F(θ) and $\nabla F^{*}\left(\eta \right)$ are reciprocal to each other. The function (R,F(θ)) is not of a Legendre type since it is not differentiable at θ=0, but the functions $\left(R_{>0},F\left(\theta \right)\right)$ and $\left(R_{>0},F^{*}\left(\eta \right)\right)$ form a Legendre-type pair when restricted to the positive domain $R_{>0}$: $F,F^{*}\in \Gamma_{1}\left(R_{>0}\right)$.*

## References

[1] Bauschke H.H., Lucet Y. (2012). What is… a Fenchel conjugate?. Not. AMS.

[2] Legendre A.M. (1805). Nouvelles méthodes pour la détermination des orbites des comètes.

[3] Hamilton W.R. (1834). On a General Method in Dynamics. Philosophical Transactions of the Royal Society of London.

[4] Gibbs J.W. (1902). Elementary Principles of Statistical Mechanics.

[5] Callen H.B. (1985). Thermodynamics and an Introduction to Thermostatistics.

[6] Weinberg S. (1995). The Quantum Theory of Fields.

[7] Mas-Colell A., Whinston M.D., Green J.R. (1995). Microeconomic Theory.

[8] Rockafellar R.T. (1970). Convex Analysis. Princet. Math. Ser..

[9] Correa R., Hantoute A., López M.A. (2023). Fundamentals of Convex Analysis and Optimization.

[10] Möllenhoff T., Khan M.E. (2023). SAM as an Optimal Relaxation of Bayes. Proceedings of the The Eleventh International Conference on Learning Representations.

[11] Artstein-Avidan S., Milman V. (2007). A characterization of the concept of duality. Electron. Res. Announc..

[12] Artstein-Avidan S., Milman V. (2009). The concept of duality in convex analysis, and the characterization of the Legendre transform. Ann. Math..

[13] Fenchel W. (1949). On Conjugate Convex Functions. Can. J. Math..

[14] Fenchel W. (2013). On conjugate convex functions. Traces and Emergence of Nonlinear Programming.

[15] Böröczky K.J., Schneider R. (2008). A characterization of the duality mapping for convex bodies. Geom. Funct. Anal..

[16] Amari S.i. (2016). Information Geometry and Its Applications.

[17] Ay N., Jost J., Vân Lê H., Schwachhöfer L. (2017). Information Geometry.

[18] Rockafellar R.T. (1967). Conjugates and Legendre transforms of convex functions. Can. J. Math..

[19] Shima H. (2007). The Geometry of Hessian Structures.

[20] Amari S.i., Nagaoka H. (2000). Methods of Information Geometry.

[21] Morales P.A., Korbel J., Rosas F.E. (2023). Geometric structures induced by deformations of the Legendre transform. Entropy.

[22] Leok M., Zhang J. (2017). Connecting information geometry and geometric mechanics. Entropy.

[23] Naudts J., Zhang J. (2024). Legendre duality: From thermodynamics to information geometry. Inf. Geom..

[24] Attouch H., Wets R.J.B. (1986). Isometries for the Legendre-Fenchel transform. Trans. Am. Math. Soc..

[25] Nock R., Nielsen F., Amari S.I. (2015). On conformal divergences and their population minimizers. IEEE Trans. Inf. Theory.

[26] Nielsen F., Nock R. (2009). The dual Voronoi diagrams with respect to representational Bregman divergences. Proceedings of the Sixth International Symposium on Voronoi Diagrams (ISVD).

[27] Amari S.i. (2009). *α*-Divergence is unique, belonging to both *f*-divergence and Bregman divergence classes. IEEE Trans. Inf. Theory.

[28] Csiszar I. (1991). Why least squares and maximum entropy? An axiomatic approach to inference for linear inverse problems. Ann. Stat..

[29] Murata N., Takenouchi T., Kanamori T., Eguchi S. (2004). Information geometry of *U*-Boost and Bregman divergence. Neural Comput..

[30] Hennequin R., David B., Badeau R. (2010). Beta-divergence as a subclass of Bregman divergence. IEEE Signal Process. Lett..

[31] Dikmen O., Yang Z., Oja E. (2014). Learning the information divergence. IEEE Trans. Pattern Anal. Mach. Intell..

[32] Barndorff-Nielsen O. (2014). Information and Exponential Families: In Statistical Theory.

[33] Nielsen F., Nock R. (2010). Entropies and cross-entropies of exponential families. Proceedings of the 2010 IEEE International Conference on Image Processing.

[34] Nielsen F., Hadjeres G. (2018). Monte Carlo information-geometric structures. Geometric Structures of Information.

[35] Graczyk P., Ishi H., Mamane S. (2019). Wishart exponential families on cones related to tridiagonal matrices. Ann. Inst. Stat. Math..

[36] Massieu F. (1876). Thermodynamique: Mémoire sur les Fonctions Caractéristiques des Divers Fluides et sur la Théorie des Vapeurs.

[37] Güler O. (1996). Barrier functions in interior point methods. Math. Oper. Res..

[38] Fujita H. (2024). The generalized Pythagorean theorem on the compactifications of certain dually flat spaces via toric geometry. Inf. Geom..

